# Log-Spiral Keypoint: A Robust Approach toward Image Patch Matching

**DOI:** 10.1155/2015/457495

**Published:** 2015-05-05

**Authors:** Kangho Paek, Min Yao, Zhongwei Liu, Hun Kim

**Affiliations:** School of Computer Science and Technology, Zhejiang University, Hangzhou, Zhejiang 310027, China

## Abstract

Matching of keypoints across image patches forms the basis of computer vision applications, such as object detection, recognition, and tracking in real-world images. Most of keypoint methods are mainly used to match the high-resolution images, which always utilize an image pyramid for multiscale keypoint detection. In this paper, we propose a novel keypoint method to improve the matching performance of image patches with the low-resolution and small size. The location, scale, and orientation of keypoints are directly estimated from an original image patch using a Log-Spiral sampling pattern for keypoint detection without consideration of image pyramid. A Log-Spiral sampling pattern for keypoint description and two bit-generated functions are designed for generating a binary descriptor. Extensive experiments show that the proposed method is more effective and robust than existing binary-based methods for image patch matching.

## 1. Introduction

Image matching based on sparse number of keypoints has been intensively studied in the community of computer vision. The state-of-the-art methods to generate keypoints with invariance to all possible image transformations are proposed in literatures. Most methods of FAST-based keypoint detection and binary-based keypoint description (such as ORB, BRISK, and FREAK) focus on fast matching of high-resolution images. In contrast, the focus of this paper is the robust matching of image patches (salient region or object) observed from the real-world images. How to improve the robust matching of image patches with low-resolution and small size has been a concerned problem in the community of computer vision due to the recent increasing application such as mobile video device, wireless sensor network, and the Internet of things. If the keypoint based mobile device whose built-in video camera captures images of low resolution is placed in a more complex environment, more advanced keypoint algorithms are necessary for the mobile device to carry out its scene and landmark recognition tasks. In addition, it is widely used in different applications, such as detection and recognition of objects in Cluttered Scenes [1, 2], matching two images with different resolutions [3], and visual saliency detection based on region descriptors [4].

A method to evaluate the performance of SIFT [5], CSIFT [6], and SURF [7] descriptors on very low-resolution images is firstly proposed [8]. Although the usefulness and weakness of SIFT and its variants in low-resolution image matching were analyzed, a method to enhance the matching performance of image patches was not presented. Also SIFT and its variants remain computationally too expensive for real-time applications [9].

Recently, to improve the efficiency of computation, some scientists proposed faster and more robust keypoint methods which combine FAST corner with binary descriptor. The Oriented Fast and Rotated BRIEF (ORB) [10], the Binary Robust Invariant Scalable Keypoints (BRISK) [11], and Fast Retina Keypoint (FREAK) [9] are good examples. FAST and its variants [12, 13] are widely used because of their low computational consumption. However, FAST detector does not produce multiscale corner keypoints. ORB detected the multiscale corner keypoints filtered by Harris at each octave layer of the image pyramid. BRISK estimated the location and the scale of each keypoint in the continuous domain via quadratic function fitting after detecting the multiscale corner keypoints in octave layers of the image pyramid as well as in layers in-between. On the other hand, to generate the bit-string of descriptor, BRISK used a sampling pattern consisting of points lying on appropriate scaled concentric circles. According to the experimental results that the size change of Gaussian kernels and the overlap of receptive fields lead to better performance, Alahi et al. in [9] designed a sampling pattern for FREAK which has the exponential change in size and the overlapping receptive fields. Since the subject of their works was the high-speed matching of high-resolution and large images, they did not pay attention to the robust matching of image patches with the low resolution and small size. In particular, multiscale keypoint detection based on image pyramid and binary descriptor generation based on sampling pattern with concentric circle structure did not have a significant effect on image patch matching.

In this paper, we propose a novel keypoint method which combines FAST corner with binary descriptor. We analyze the performance of keypoint detection method based on image pyramid via some experiments for image patch matching, which reveals that they are not suitable to match image patches. To alleviate such weakness, we present a keypoint detector to enhance the matching performance of image patches. Our detector estimates the location, scale, and orientation of keypoints using the Log-Spiral sampling pattern for keypoint detection without consideration of image pyramid. Then we obtain a corresponding relationship for adjusting the number of sampling points in the keypoint detection sampling pattern with response to the size change of image patch. Moreover, we present a new binary descriptor to enhance the performance of current binary descriptors. Our descriptor relies on the Log-Spiral sampling pattern for keypoint description to capture more information around a keypoint and two bit-generated functions to enhance the independence between descriptors. We compare the accuracy of the image patch matching against two state-of the-art methods using a lot of low-resolution and real-world images. Our method outperforms all of binary-based methods in terms of recall and number of correct matches.

## 2. LOS-K: The Proposed Method

Our method is built on Log-Spiral (logarithmic-spiral) model. For this reason we call it LOS-K (Log-Spiral keypoint). In this section, we describe the key stages in LOS-K, namely, keypoint detection and description.

### 2.1. Keypoint Detection

#### 2.1.1. Image Pyramid and Image Patch Matching

The multiscale corner keypoints are detected from each octave image in image pyramid. Hence, the number of octave layers in image pyramid has a great effect on performance of image matching. We here utilize BRISK, a state-of the-art method, to analyze the effect of this parameter on the performance of image patch matching. To obtain the test image patches, we reduce the standard images provided by Mikolajczyk et al. in [14, 15]. The patch dataset consists of the 3 subsets (Bikes, Boat, and Graffiti) and each subset includes 3 pairs of patches with different resolution (90 × 70, 180 × 140, and 270 × 210).

The number of correct matches determines the accuracy of image matching. We use the number of correct matches as a metric. We carry out our experiments by MATLAB R2014b. The MATLAB function estimateGeometricTransform is used to compute the number of correct matches. The function detectBRISKFeatures is used to detect the keypoints and extractFeatures is used to generate the binary descriptors of keypoints. Keypoint matching is performed by a function matchFeatures. The number of octave layers in image pyramid can be varied by tuning the parameter NumOctave in function detectBRISKFeatures. In order to obtain all possible matches, we set the parameter MaxRadio in matchFeature to 0.9.

**Figure 1:**
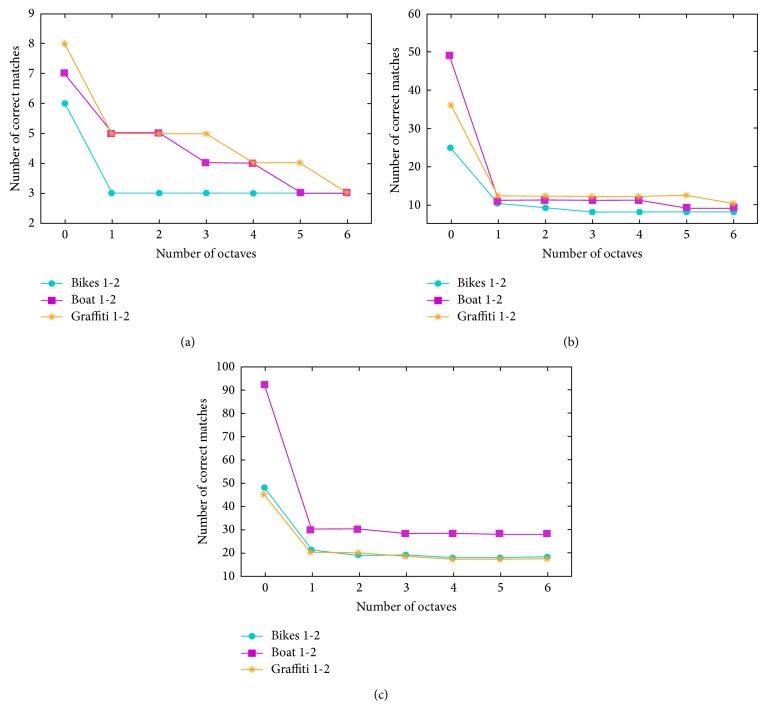
The change of number of correct matches with increasing of number of octaves: (a) matching test in a subset that contains 90 × 70 image patches, (b) 180 × 140 image patches, and (c) 270 × 210 image patches.

**Figure 2:**
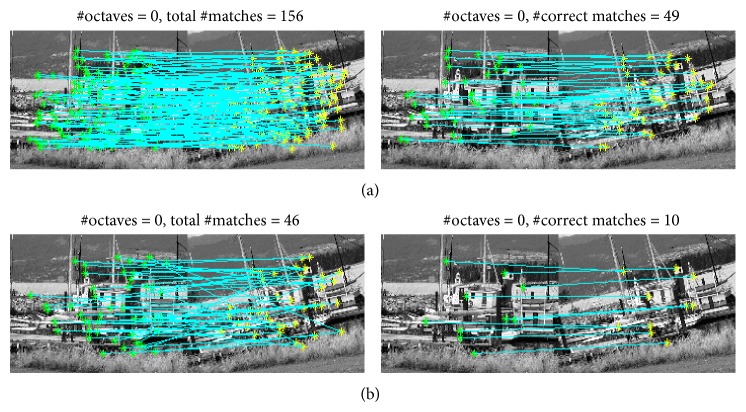
Matching samples on 180 × 140 Boat patches 1 and 2: (a) when #octave = 0 and (b) when #octave = 3.

We compute the number of correct matches with increasing the number of octave layers. Experiment results in Figure 1 show that when the number of octave layers is zero (when image pyramid is not employed), the number of correct matches clearly increases. For keypoint detection, a maxima-search using the FAST score is performed in the image space. The keypoints do not have the scale components. Hence a constant value is adopted as common scale component for descriptor generation. It is difficult to guarantee the invariance to scale which is crucial for high-quality keypoints. Increasing the number of octave layers, the BRISK detector performs the searching for maxima not only in the image plane but also in scale-space using the FAST score and detects the limited number of multiscale corner keypoints with a different scale component. However, experimental results show that as the number of octave layers increases, the number of correct matches decreases; for example, in Figure 2, for #octave = 0 (the number of octave layers is zero), #correct matches = 56 (the number of correct matches is 56); for #octave = 3, #correct matches  =  10. These results demonstrate that when the images to be matched are smaller, the keypoint detection method based on image pyramid could not correctly estimate the location and scale of keypoints.

To alleviate the deficiencies of pyramid method, in next section we introduce a method to detect the multiscale corner keypoints directly from original image patch without consideration of image pyramid.

#### 2.1.2. Keypoint Detection Using Log-Spiral Sampling Pattern

*(A) Log-Spiral Model*. The Log-Spiral sampling pattern is built on Log-Spiral model. On the two-dimensional polar coordinate system (*r*, *ϕ*), the Log-Spirals are defined by the following:

(1) $$\begin{array}{c} r=ae^{b\phi }, \end{array}$$

where *ϕ* ∈ *R* indicates an angle variable, *r* ∈ *R* indicates a radius variable which means distance from the origin, and *a* > 0, *b* are constant real numbers. *a* is arbitrary constant controlling the position of point where spiral begins. In case of *b* > 0, formula (1) describes a curve which diverges at the origin as *ϕ* increases. In case of *b* < 0, formula (1) describes a curve which converges at the origin as *ϕ* increases. On the two-dimensional Cartesian coordinate system, the Log-Spiral model (1) is rewritten as follows:

(2) $$\begin{array}{c} x=a\exp\left(b\left(\phi +\phi_{0}\right)\right)\cos\left(\phi +\phi_{0}\right),\\ y=a\exp\left(b\left(\phi +\phi_{0}\right)\right)\sin\left(\phi +\phi_{0}\right), \end{array}$$

where *ϕ*_0_ is a constant which indicates the initial angle when *ϕ* = 0.

To apply Log-Spiral to image mapping, the discrete form of Log-Spiral model needs to be introduced; it is as follows:

(3) $$\begin{array}{c} x_{k}=a\exp\left(b\left(k\Delta \phi +\phi_{0}\right)\right)\cos\left(k\Delta \phi +\phi_{0}\right)+x_{0},\\ y_{k}=a\exp\left(b\left(k\Delta \phi +\phi_{0}\right)\right)\sin\left(k\Delta \phi +\phi_{0}\right)+y_{0},\\ \Delta \phi =\frac{2\pi }{n_{\phi }},\;\;n_{r}=\frac{N}{n_{\phi }}, \end{array}$$

where Δ*ϕ* is an angle increment, *k* = 1,…, *N*, *N* is the total number of sampling points on the discrete Log-Spiral curve, and *n*_*ϕ*_ is the number of sampling points within the 2*π* radian on the curve.

Given an image *Ι*, the Log-Spiral mapping *Ι*(*x*, *y*) ↦ Sp(*θ*) is as follows:

(4) $$\begin{array}{c} \text{Sp}\left(k\right)=Ι\left(x_{k},y_{k}\right), \end{array}$$

where Sp is a one-dimensional transformed vector.

*(B) Log-Spiral Sampling Pattern for Keypoint Detection*. We here introduce a Log-Spiral sampling pattern for the multiscale corner keypoint detection from original image. FAST uses Bresenham's circle of diameter 7 pixels as test mask; 16 pixels on a circle have to be compared to the value of the nucleus. In the case of FAST-9, the criteria for a pixel to be a corner must be at least 9 connected pixels on the circle which are brighter or darker than a threshold determined by the center pixel value. To detect the multiscale corner keypoints, *n*_*s*_ of octave images with different resolution (downsampled by a factor of 2) and Bresenham's circle of 16 pixels are universally used. In contrast, our method relies on the premise that multiscale corner keypoints could be detected from an original image using *n*_*s*_ of multiscale discrete circles.

We define the multiscale discrete circles of which individual diameter satisfy formula (5). Each multiscale discrete circle consists of 16 sampling points:

(5) $$\begin{array}{c} d_{t}=7\cdot 2^{t},\;\left\{t=0,\ldots ,n_{s}-1\right\}, \end{array}$$

where *t* is a scale factor. To design a Log-Spiral sampling pattern which replaces typical image pyramid consisting of 4 octave layers, we set *n*_*s*_ to 4.

**Figure 3:**
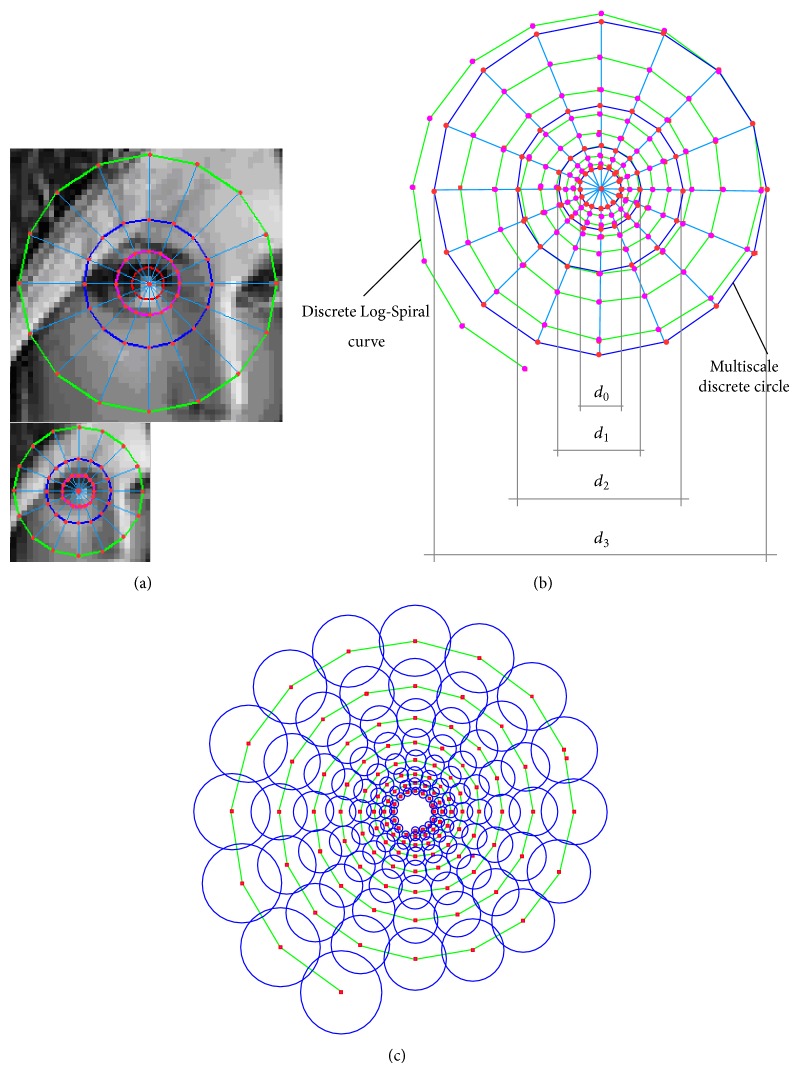
Conceptualization of Log-Spiral sampling pattern for the multiscale corner keypoint detection: (a) the image pyramid and the multiscale discrete circles, (b) the comparison of sampling point distribution on 4 multiscale discrete circles and a discrete Log-Spiral curve, and (c) the Log-Spiral sampling pattern used to detect the multiscale corner keypoints.

Figure 3(a) (up) shows the 4 concentric multiscale discrete circles. Spacing angle between two neighboring sampling points on all multiscale discrete circles is the same. When *t* = 0, a red discrete circle of diameter 7 pixels becomes Bresenham's circle and it is used as test mask on an original image. When *t* = 1, a pink discrete circle of diameter 14 pixels in Figure 3(a) (up) becomes Bresenham's circle of diameter 7 pixels on first octave image (Figure 3(a) (down)). Note that although the downsampling operation changes the size of multiscale discrete circles, it does not change the structural relationship between the intensity values of sampling points on a circle. Considering the suitable smoothing operation at each sampling point, we conclude that the multiscale corner keypoints with coarser scale could be detected from an original image using multiscale discrete circles as test masks. Although this method can obtain the correct location of keypoints, it cannot detect the multiscale corner keypoints with true scale as it does not fully capture the local image information around a keypoint.  Figure 3(b) shows the comparison of sampling point distribution on 4 multiscale discrete circles and a discrete Log-Spiral curve. As illustrated in Figure 3(b), 64 sampling points on all multiscale discrete circles can only capture the image information in local regions around circles corresponding to 4 specific diameter parameters, while, for the same local image region, 128 sampling points on a discrete Log-Spiral curve can capture more image information. These sampling points enable the detection of high-quality keypoints from low-resolution image patches.

Figure 3(c) shows the Log-Spiral sampling pattern with *N* = 128 points, which is used to detect multiscale corner keypoints. The positions of points on the sampling pattern are obtained by setting of the following parameters about formula (3): *a* = 3.5, *ϕ*_0_ = −*π*/2 (to match the positions of two sampling points (a sampling point where spiral begins and a sampling point of first multiscale discrete circle)); *b* = 0.047, *n*_*r*_ = 8 (to obtain the equidistribution of sampling points within an annulus between two neighboring multiscale discrete circles); *n*_*θ*_ = 16 (to match spacing angle between two neighboring points on a multiscale discrete circle and a spiral curve). The intensity value of each sampling point, Sp(*k*), *k* = 1,…, 128, is the smoothed intensity by pixels belonging to the circular region of sampling point, where the circle's radius increases as the distance from the location of keypoint increases. For the consideration of the speed of computation, smoothing based on integral image is applied. Smoothing operation reduces the effects of noise and lighting changes.

*(C) Keypoint Detection Algorithm Using Log-Spiral Sampling Pattern*.* Firstly*, obtain the locations of keypoint candidates using FAST-9 algorithm and remove the candidates with lower score using 8 neighboring search based on FAST score.

*Secondly*, construct the Log-Spiral sampling pattern at each candidate and calculate one-dimensional vector Sp(*i*)  {*i* = 1,…, 128}.

*Thirdly*, detect the multiscale corner keypoints and confirm their locations. Initially, obtain new FAST score of each candidate using intensity values of sampling points belonging to Sp. Under the assumption that a vector Sp is composed of 128 components, obtain 112 segments from Sp. Each segment is composed of 16 components, such as {Sp(1),…, Sp(16)}, {Sp(2),…, Sp(17)},…. Using each segment as test mask, obtain 112 FAST scores and set maximum score to FAST score of each candidate. However, this method requires more time. According to the experimental results that good corner features are repeatedly detected at different scales, we divided Sp into eight segments:

(6) X1∈Sp1,…,Sp16,X2∈Sp17,…,Sp32,…,X8∈Sp113,…,Sp128.

Next, for each segment *X*_*j*_  {*j* = 1,…, 8}, obtain FAST score *S*_*i*_  {*i* = 1,…, 8}. Under the assumption that *M* candidates are detected, we construct a score matrix *S* with *M* × 8 in size. Also, set a maximum score in each row of score matrix to a score of each candidate and obtain a score vector *V* with *M* × 1 in size. Order a score vector *V* from high score to low one and remove all candidates with low scores within scale neighbor of candidate with high score using greedy algorithm. Ultimately, adopt remaining candidates as multiscale corner keypoints and confirm the location of each keypoint.

*Finally*, determine the scale of each keypoint. Considering relationship (5) between scale and multiscale discrete circle diameter, finer spacing of *d*_*t*_ will divide scale-axis into the finer intervals. 128 sampling points on Log-Spiral sampling pattern divide the diameter range between *d*_0_ and *d*_3_ into 128 finer intervals. Hence, the scale estimation method based on a diameter value of sampling point would be able to obtain the finer and true scale. Also, FAST score is defined as the maximum threshold still considering an image point a corner. A segment *X*_*i*_ producing a maximum score becomes a very robust annulus around a keypoint for several transformations. Therefore, we estimate the true scale of each keypoint using sampling points within a segment *X*_*i*_. Initially, find a segment *X*_*i*_ producing a maximum score in each row of score matrix and find 9 connected sampling points on a segment *X*_*i*_ which are brighter or darker than a threshold determined by the center pixel value. Next, obtain a scale of each keypoint by formula (5) with the diameter value of central sampling point among 9 sampling points.

*(D) Dominant-Orientation of Keypoint*. To improve the rotation invariance, we apply a simple but reliable estimation of keypoint orientation. The Dominant-Orientation is also estimated by using the segment *X*_*i*_ producing maximum score. Let us suppose an annulus using radii of primary and last components in *X*_*i*_ as radii of inner and outer circles, respectively. Then, divide equally an annulus into 36 sectors in the rotational direction and calculate the absolute value of difference between average intensity values of two sectors located symmetrically. Continually, select the symmetric sectors producing maximum one among 18 differences and determine a rotational angle of sector producing larger average intensity value as a Dominant-Orientation of each keypoint.

### 2.2. Keypoint Description

#### 2.2.1. Log-Spiral Sampling Pattern for Keypoint Description

Based on the experimental results that changing the size of the Gaussian kernels with respect to the log-polar retinal pattern leads to better performance and overlapping the receptive fields increases the performance, the FREAK designed a retinal sampling pattern which has the exponential change in size and the overlapping receptive fields. However, the FREAK sampling pattern also employed the arrangement of sampling points on the concentric circles similar to BRISK. Thereby it does not capture more information in annulus region between two neighboring concentric circles. We here introduce an improved retinal sampling pattern, named Log-Spiral sampling pattern for keypoint description. The Log-Spiral sampling pattern is built on a discrete Log-Spiral curve. As mentioned above, the Log-Spiral sampling pattern can capture more information around a keypoint than others.

**Figure 4:**
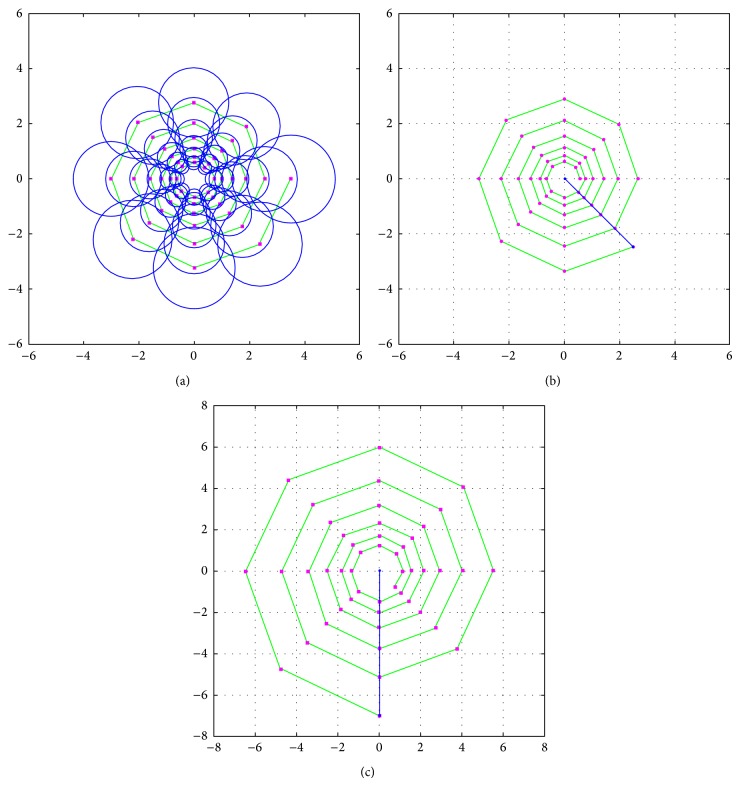
The Log-Spiral sampling pattern with *N* = 48 points. The small pink circles denote the positions of sampling points; the bigger, blue circles are drawn at a radius *σ* corresponding to the standard deviation of the Gaussian kernel used to smooth the intensity values at the receptive fields on the pattern. (a) Standard sampling pattern (*t* = 1, *ϕ*_0_ = 0). (b) Sampling pattern rotated (*t* = 1, *ϕ*_0_ = *π*/4). (c) Sampling pattern rotated and resized (*t* = 2, *ϕ*_0_ = *π*/2).

Figure 4(a) shows a standard Log-Spiral sampling pattern used for generation of binary descriptor. A discrete Log-Spiral curve (with *N* = 48 points) which converges at the origin as angle variable increases is used in our sampling pattern. The position of each sampling point satisfies the following:

(7) $$\begin{array}{c} x_{k}=a\exp\left(-0.05\left(0.785k+\phi_{0}\right)\right)\cos\left(0.785k+\phi_{0}\right)+x_{0},\\ y_{k}=a\exp\left(-0.05\left(0.785k+\phi_{0}\right)\right)\sin\left(0.785k+\phi_{0}\right)+y_{0}, \end{array}$$

where *k* = 1,…, *N*, *x*_0_ = *y*_0_ = 0, *a* = 3.5, and *ϕ*_0_ = 0. The intensity value of each sampling point, Sp(*k*), is the smoothed intensity by pixels belonging to the circular region of sampling point, where the circle's radius increases as the distance from the location of keypoint increases. For speed of computation, smoothing operation based on integral image is applied. Smoothing operation reduces the effects of noise and lighting changes.

For the formation of the rotation- and scale-normalized descriptor, the sampling pattern should be resized by scale factor and rotated by Dominant-Orientation around a keypoint. For this, we change two parameters *a* and *ϕ*_0_ in formula (7), which indicate the radial and angular coordinates of sampling point where spiral begins. In order to change the size of sampling pattern, we adopt *a* = 3.5 × *t* (*t* is the scale of each keypoint). In order to rotate the sampling pattern, we adopt a Dominant-Orientation of each keypoint as *ϕ*_0_. In practical application, the center point of sampling pattern (*x*_0_, *y*_0_) denotes the location of each keypoint. Figures 4(b) and 4(c) show two examples of sampling patterns.

#### 2.2.2. Building the Binary Descriptor

The FAST detects two kinds of corners, light and dark corners. Our detector uses FAST corners as keypoint candidates. Hence, keypoints detected would contain the light and dark ones.

The distinction between retinal cells is their behavior in response to sudden steps of light in their receptive field. Some cells respond strongly to light increments, while others respond strongly to light decrements. The first is termed “ON” cells, and the second is termed “OFF” cells. The spatial center-surround opposition of ON- and OFF-ganglion cells is well approximated by a Difference of Gaussian (DoG), whereas the signs of ON- and OFF-DoGs are opposite. Taking into account the response characteristics of retinal ON- and OFF-ganglion cells, we introduce two bit-generated functions, *F*_*off*⁡_(*P*_*k*_) and *F*_*on*⁡_(*P*_*k*_).

The binary descriptor is generated by threshold the difference between pairs of receptive fields with their corresponding Gaussian kernel. A binary string generated by a sequence of one-bit Difference of Gaussians (DoG), *B*, is defined as follows:

(8) $$\begin{array}{c} B=\sum_{0\leq k<M}2^{k}F\left(P_{k}\right), \end{array}$$

where *P*_*k*_ is a pair of receptive fields in the Log-Spiral sampling pattern and *M* is the descriptor size.

Since a dark-Keypoint corresponds to a sudden step of light with dark-center and bright-surround, an OFF-ganglion cell represents a good response to dark-Keypoint. For this reason we call the binary descriptor of dark-Keypoint OFF-descriptor. At this time, bit-generated function of OFF-descriptor *F*_*off*⁡_(*P*_*k*_) is defined as follows:

(9) $$\begin{array}{c} F_{\mathrm{off}}\left(P_{k}\right)=\left\{\begin{array}{cc} 1 & \text{if}\;\left(\text{Sp}\left(P_{k}^{r_{1}}\right)-\text{Sp}\left(P_{k}^{r_{2}}\right)\right)>0\\ 0 & \text{otherwise}, \end{array}\right. \end{array}$$

where Sp(*P*_*k*_^*r*_1_^) is the smoothed intensity of the first receptive field in the pair *P*_*k*_ and Sp(*P*_*k*_^*r*_2_^) is the smoothed intensity of the second receptive field. *P*_*k*_ should be selected from periphery of the sampling pattern to center; *r*_1_ > *r*_2_, *r*_1_ = *N*,…, 2, and *r*_2_ = *r*_1_ − 1,…, 1. In case of light-Keypoint, bit-generated function of ON-descriptor *F*_*on*⁡_(*P*_*k*_) is defined as follows:

(10) $$\begin{array}{c} F_{\mathrm{on}}\left(P_{k}\right)=\left\{\begin{array}{cc} 1 & \text{if}\;\left(\text{Sp}\left(P_{k}^{r_{1}}\right)-\text{Sp}\left(P_{k}^{r_{2}}\right)\right)>0\\ 0 & \text{otherwise}. \end{array}\right. \end{array}$$

*P*_*k*_ should be selected from center of the sampling pattern to periphery; *r*_1_ < *r*_2_, *r*_1_ = 1,…, *N* − 1, and *r*_2_ = *r*_1_ + 1,…, *N*. The OFF- and ON-descriptors originating from the same image have their own distinctiveness, so the robustness of descriptors would be increased.

## 3. Experiments and Results

Three experiments to evaluate the performance of the proposed method are discussed here as follows: (i) the setting of corresponding relationship between the size of image patch and the number of sampling points in the Log-Spiral sampling pattern for keypoint detection, (ii) verification of the efficiency of Log-Spiral sampling pattern for keypoint detection and testing of viewpoint-, scale-, and rotation-invariance using standard image patch dataset, and (iii) evaluation and comparison of the overall LOS-K algorithm using real-world patch dataset.

### 3.1. Experiment  1

The size of sampling pattern for the generation of a keypoint descriptor is adjusted by the scale component of keypoint. If the size of this sampling pattern is larger than the size of original image patch, it is self-evident fact that the sampling pattern cannot generate a high reliability descriptor. That is to say that a significant dependency exists between the size of image patch and the keypoint scale. One important advantage of the Log-Spiral sampling pattern for keypoint detection is that it can flexibly change the scale range of keypoints by adjusting the number of sampling points. For this reason, the problem of varying the scale range of the keypoints with correspondence to the size change of the image patch will be considered another problem to change the number of sampling points in the sampling pattern.

Prior to the performance comparison of the proposed method, we first perform an experiment to set the corresponding relationship between the size of the image patch and the number of sampling points in the Log-Spiral sampling pattern for keypoint detection.

Test image patches are made by reducing the nine standard images (the first three in each subset (Bikes, Boat, and Graffiti)) in the dataset provided by the Mikolajczyk and Schmid [14, 15]. Our dataset consists of the five patch groups: Group 1 (nine of 90 × 70 image patches), Group 2 (180 × 140), Group 3 (270 × 210), Group 4 (360 × 280), and Group 5 (450 × 350). Each group is composed of the three subgroups. Each of the subgroups contains three image patches exhibiting an increasing amount of transformation. The transformations cover view-point change (Graffiti), zoom and rotation (Boat), and blur (Bikes). All comparisons here are performed for the first image patch in each subgroup. The number of sampling points to be tested is as follows: *N* = 16,32,48,64,80,96,112,128.

The main parameters of the LOS-K are the detection and matching thresholds. The LOS-K detection threshold is the minimum intensity difference between a corner and its surrounding region. For matching of two descriptors, the LOS-K uses a fast algorithm proposed by Muja and Lowe in [16]. The matching threshold is nearest neighbor ratio between two descriptors. The LOS-K varies these two parameters *T*_1_ ∈ {0.3,0.4,0.5,0.6} and *T*_2_ ∈ {0.6,0.7,0.8,0.9} and runs for each combination of both parameters. When *T*_1_ and *T*_2_ values increase, the number of keypoints detected decreases while the number of matches increases.

**Figure 5:**
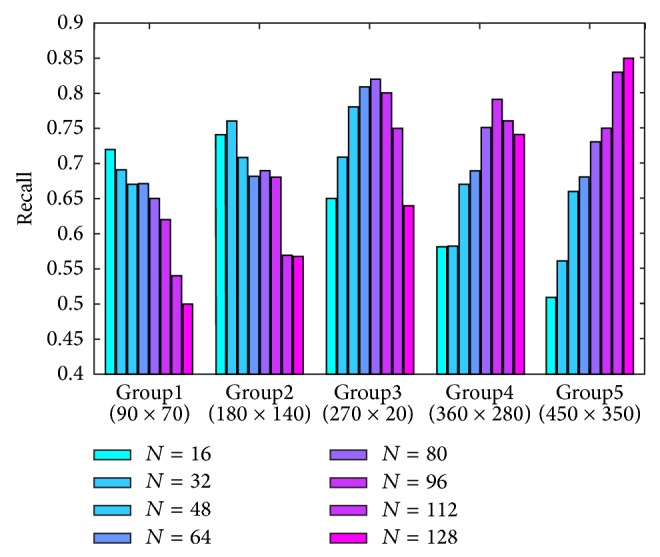
Recall test for setting of corresponding relationship between the size of image patch and the number of sampling points in the Log-Spiral sampling pattern for keypoint detection.

In order to obtain the corresponding relationship, we calculate the average recall value of each group according to the change of number of sampling points. A selection of results (*T*_1_ = 0.5, *T*_2_ = 0.7) is shown in Figure 5. The experimental results clearly show that there is an approximate proportional relation between the size of image patch and the number of sampling points. Namely, the experiments show that better matching results can be obtained when detecting the keypoints using the sampling pattern with small sampling points for small image patches and the matching performance can be improved when increasing the number of sampling points as patch size increases. Experiments for different values of *T*_1_ and *T*_2_ also show the same matching results.

According to the above analysis, we obtained a table of corresponding relationship between the size of image patch and the number of sampling points in the Log-Spiral sampling pattern for keypoint detection. In Table 1, the patch size indicates the total number of pixels within image patch.

### 3.2. Experiment  2

In this experiment, we verify the efficiency of Log-Spiral sampling pattern for keypoint detection and test the viewpoint-, scale-, and rotation-invariance using standard image patch dataset. Image patches are made by reducing the 36 standard images (Bikes, Boat, Graffiti, Wall, Leuven, and Ubc) in the dataset provided by the Mikolajczyk and Schmid [15, 16]. Our dataset consists of the five patch groups: Group 1 (36 of 90 × 70 image patches), Group 2 (180  ×  140), Group 3 (270 × 210), Group 4 (360 × 280), and Group 5 (450 × 350). Each group is composed of the six subgroups which contains six image patches exhibiting an increasing amount of transformation. The transformations cover view-point change, zoom and rotation, and blur. All comparisons here are performed for the first image patch in each subgroup.

Two combinations of detector/descriptor are used for performance comparison: BRISK/BRISK and BRISK/FREAK. For the sake of consistency with results presented in other works, we use the following keypoint detector, descriptor, and matching functions in the MATLAB platform: detectBRISKFeatures, extractFeatures, and matchFeatures. The function detectBRISKFeatures is used to detect the BRISK keypoints and extractFeatures is used to generate the binary descriptors of BRISK and FREAK. Descriptor matching is performed by function matchFeatures. For the detection and description of keypoints, we use all of the default parameters supplied by the programs except for the number of octave layers. For each image group, the LOS-K uses the different Log-Spiral sampling patterns for keypoint detection that satisfies the corresponding relationship shown in Table 2. The LOS-K also uses the function matchFeatures for descriptor matching. Both of BRISK and LOS-K detectors use corner points detected by FAST detector as candidates of keypoints, while BRISK, FREAK, and LOS-K use the same MATLAB matching function for descriptor matching. Hence, we use the same detection and matching thresholds in all algorithms.

**Figure 6:**
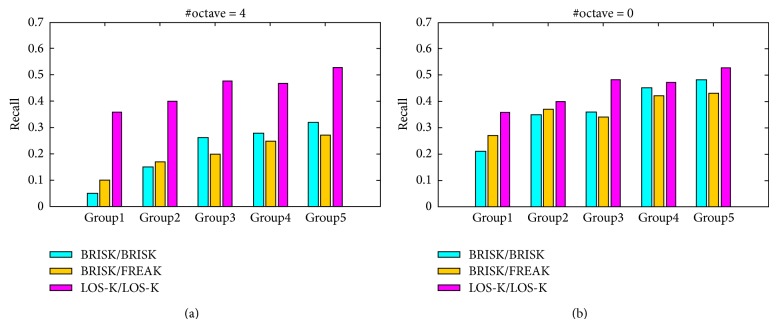
Average recall comparison of the three algorithms for five image groups. Each vertical bar in the plots corresponds to average recall value for each image group. (a) Comparison of the matching results obtained when BRISK perform the keypoint detection using the image pyramid consisting of 4 octave layers and (b) when the number of octave layer set to 0.

Figure 6 summarizes the performance of each algorithm in terms of average recall value of each group and shows a selection of results (detection threshold = 0.2, matching threshold = 0.9).  Figure 6(a) shows a comparison of matching results (BRISK detect the keypoints using an image pyramid and LOS-K detect the keypoints using a sampling pattern based on the corresponding relationship shown in Table 2). As illustrated in Figure 6(a), the recall values of both BRISK and FREAK decrease quickly with the decrease of image patch size, especially, when the patch size is smaller than 180  ×  140 pixels. This reveals that the image pyramid based multiscale keypoint detection method is not an effective way to match the image patches with a small size and low resolution. The location and scale of keypoint estimated by using low-resolution images of the image pyramid do not have high accuracy, which leads to the reduction of robustness of descriptors.

On the other hand, the recall values of LOS-K decrease less quickly. This reveals that the LOS-K is very robust with respect to the change of patch size. The major cause that the LOS-K leads to good matching results can be explained in several aspects. Firstly, the LOS-K directly performs the keypoint detection from an original image patch using Log-Spiral sampling pattern without consideration of image pyramid. In this case, it can estimate the position, scale, and orientation of keypoints with high accuracy using rich image information and generate the descriptors with high robustness.  Figure 6(b) shows that when the BRISK detector detects directly the keypoints from an original image without consideration of low-resolution images in high octave layers, the BRISK and FREAK can improve their performance in the small patch matching. However, it increases the consumption of computing resource due to the large number of keypoint detection. Secondly, the LOS-K detects the effective keypoints for matching of given image patches which depend on the corresponding relationship between the size of image patch and the number of sampling points. This has been already proved in Experiment  1. Finally, the LOS-K uses a deterministic Log-Spiral sampling pattern for keypoint description, which can capture more information around a keypoint and uses two bit-generated functions to enhance the independence between descriptors.

In this experiment, we did not perform separately the testing of viewpoint-, scale-, and rotation-invariance. However, the test dataset contains a sequence of image patches exhibiting an increasing amount of transformation. The LOS-K shows a good matching performance for these image patches.

### 3.3. Experiment  3

**Figure 7:**
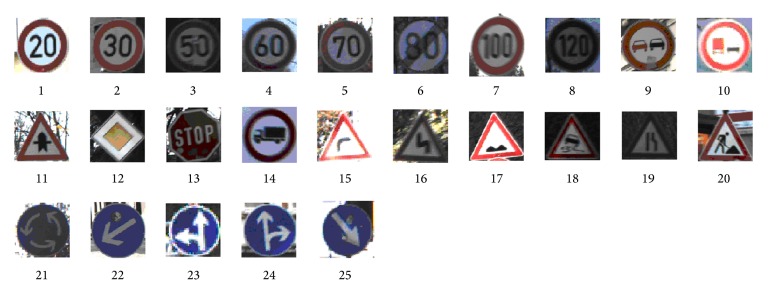
25 traffic signs are used as query patches in the detection test.

In the third experiment, we evaluate the performance of the overall LOS-K algorithm using real-world images. The dataset provided by Houben et al. in [17] comprises 900 full images containing 1206 traffic signs. All images in the dataset capture different scenarios (urban, rural, and highway) during daytime and dusk featuring various weather conditions and their resolution is 1360 × 800 pixels. Every image contains the regions of interest (ROIs) of the visible traffic signs and the specific traffic sign class (e.g., stop sign, speed limit 60, and speed limit 80). The size of traffic signs varies between 16 and 128 pixels with respect to the longer edge. It should be noted that most of the traffic sign instances occur only once in their dataset. We select 25 traffic signs whose patch size is larger than 50 × 50 pixels and perform the experiments to detect them from 900 real-world images.  Figure 7 shows 25 traffic signs.

In the keypoint based traffic sign detection, the number of correct matches between the keypoints originating from the query patch and the real-world image guarantees the accuracy of detection results. Therefore, we use the number of correct matches as a measure. The BRISK and FREAK methods are used for performance comparison. In addition, SURF method is used for comparative analysis. The matching results for low-resolution images with blurring and a change of illumination (both brightening and darkening) show the good performance of SURF [7]. The function detectSURFFeatures is used to detect the BRISK keypoints and extractFeatures is used to generate the binary descriptors of SURF. Descriptor matching is performed by a function matchFeatures. We use all of the default parameters supplied by the programs except for the number of octave layers.

In order to balance a fair comparison, we use the same detection and matching thresholds for all methods. Taking into consideration the small size of the query traffic signs, we set the number of sampling points of LOS-K detector to 16; in case of BRISK detector, set the number of octaves to 0; and in case of SURF detector, set the number of octaves and number of scale levels per octave to 0 and 4.

**Figure 8:**
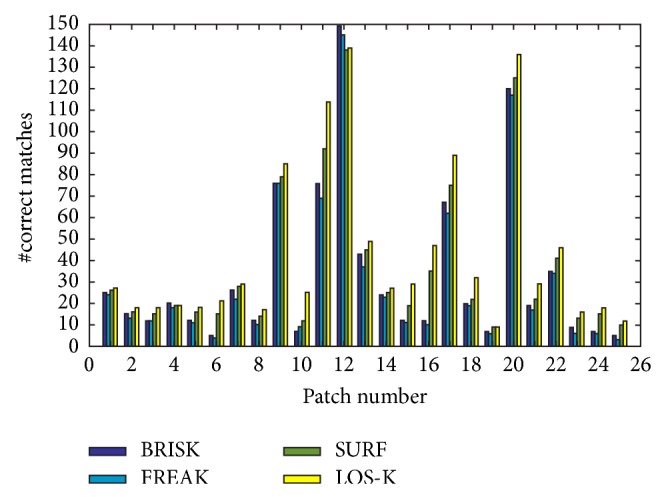
Performance comparison of each algorithm in terms of number of correct matches.

**Figure 9:**
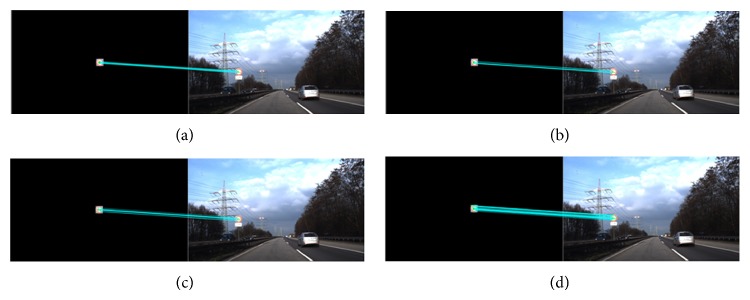
Visual comparison of the detection results for a traffic sign of patch 10. (Up) BRISK, #correct matches = 7; (middle) FREAK, #correct matches = 9; (bottom) LOS-K, #correct matches = 23.

Figure 8 summarizes the performance of each algorithm in terms of number of correct matches and shows a selection of results (detection threshold = 0.1 and matching threshold = 0.9). As shown in Figure 8, the LOS-K is able to detect the query patches from real-world images captured under various weather conditions more correctly, regardless of the type and size of the query patches.  Figure 9 shows a visual comparison of detection results obtained by three methods.

## 4. Conclusion

In this paper, we study the problem of matching the image patches with the low resolution and small size. The LOS-K detector is proposed to improve the matching performance of image patches. Our detector estimates the location, scale, and orientation of keypoints using Log-Spiral sampling pattern for keypoint detection without consideration of image pyramid. In addition, it detects the effective keypoints for matching the given image patches, which is based on the corresponding relationship between the size of image patch and the number of sampling points. Then, we present a LOS-K descriptor to enhance the performance of current binary descriptors. Our descriptor relies on the Log-Spiral sampling pattern for keypoint description and two bit-generated functions. Our evaluation results indicate that the proposed method is more effective and robust than existing binary-based methods for image patch matching. To obtain the relationship between the size of image patch and the number of sampling points, we relied only on the statistical analysis of experimental results and did not consider the relation between this problem and the spatial distribution or between this problem and the bandwidth in a frequency domain. It will be our future work in the goal, the realization, and completion constant.

## Figures and Tables

**Table 1:** Corresponding relationship between the size of image patch and the number of sampling points.

Patch size (×10^4^ pixels)	0.2~0.8	0.8~2	2~4	4~6	6~9	9~12	12~15	15~
# sampling points	16	32	48	64	80	96	112	128

**Table 2:** Corresponding relationship between the size of testing image patch and the number of sampling points.

Image patch size	90 × 70	180 × 140	270 × 210	360 × 280	450 × 350
# sampling points	16	32	64	80	112
